# Influence of AMY1 gene copy number on salivary amylase activity changes induced by exercise in young adults

**DOI:** 10.14814/phy2.70099

**Published:** 2024-10-25

**Authors:** Yui Kobayashi, Eri Koibuchi, Keishoku Sakuraba, Yoshio Suzuki

**Affiliations:** ^1^ Faculty of Human Development Department of Health and Education Kokugakuin University Yokohama Kanagawa Japan; ^2^ Graduate School of Health and Sports Science Juntendo University Inzai Chiba Japan

**Keywords:** aerobic exercise, copy number variation, excretion, interval training, secretion, stress

## Abstract

Human salivary amylase secretion increases in response to stress; the activity has been reported to rise significantly with high‐intensity exercise. The human salivary amylase gene (AMY1) has copy number variation, with the copy number correlating with salivary amylase activity. However, the relationship between individual AMY1 copy number and salivary amylase activity in response to exercise remains unclear. In this study, we investigated AMY1 copy number and fluctuations in amylase activity in 42 healthy university students (25 males and 17 females). Participants engaged in intermittent round‐trip interval training on a basketball court. Saliva samples were collected pre‐ and post‐exercise to measure amylase activity. DNA was extracted from the oral mucosa, and AMY1 copy number was quantified using RT‐PCR. Results showed a significant increase in amylase activity postexercise. Additionally, amylase activity pre‐ and post‐exercise was positively correlated with AMY1 copy number. The generalize linear model showed that the exercise‐induced increase in amylase activity per AMY1 gene was negatively related to the AMY1 copy number and aerobic fitness. Gender has no effect on amylase activity. These results suggest a different mechanism for the constitutive and exercise‐induced amylase secretion, while aerobic fitness may be independently involved in the secretion.

## 1 INTRODUCTION

Saliva—the first digestive fluid—is widely used as a noninvasive biomarker sample. In particular, salivary amylase, which accounts for the majority of salivary proteins, has been extensively studied (Granger et al., 2007). Salivary amylase secretion is influenced by the stress system, transmitted from the hypothalamus via the sympathetic nervous system to the adrenal medulla (Nater et al., 2006; van Stegeren et al., 2006), and it increases in response to physical and psychological stress (Granger et al., 2007). Our systematic review of exercise‐induced amylase activity reported a significant increase in healthy adults following high‐intensity exercise above 70% VO_2_max (Koibuchi & Suzuki, 2014). Therefore, salivary amylase activity is a valid index for the noninvasive assessment of exercise stress.

The human salivary amylase gene (AMY1) contains a salivary gland‐specific promoter in its upstream region, which includes a gamma‐actin pseudogene and an endogenous retrovirus. While mammals have multiple duplicates of the pancreatic amylase gene (AMY2), the insertion of the gamma‐actin pseudogene occurred after the divergence of New World monkeys from the primate lineage, and the retrovirus insertion occurred after the divergence of Old World monkeys (Samuelson et al., 1996). In humans, the AMY1 gene has copy number variations. Among great apes, chimpanzees (*Pan troglodytes*) lack copy number polymorphisms, while the bonobos (*Pan paniscus*) have such polymorphisms, through the genes may lose functionality due to coding region disruptions (Perry et al., 2007). Notably, only humans have multiple copies of functional salivary amylase genes. Therefore, in humans, salivary amylase activity is also influenced by the copy number of the salivary AMY1 (Alberti et al., 2015; Falchi et al., 2014).

At present, salivary amylase activity is widely used as a noninvasive marker for psychological (Man et al., 2023; Mishra & Rašticová, 2024; Shah et al., 2024; Špiljak et al., 2022) and physical (Kamarauskas & Conte, 2022; Thieux et al., 2024) stresses. However, the impact of AMY1 copy number on changes in salivary amylase activity in response to exercise has not been reported. Therefore, this study aimed to investigate the effect of AMY1 copy number on exercise‐induced transient changes in amylase activity. This study demonstrated a positive correlation between salivary amylase activity and AMY1 copy number pre‐ and post‐exercise, while the exercise‐induced increase in activity was largely independent of AMY1 copy number. In addition, the change in amylase activity per AMY1 gene was negatively correlated with AMY1 copy number and aerobic fitness.

## 2 METHODS

### 2.1 Participants

This study included 42 university students (25 males and 17 females) (Table 1). Before recruitment, the experimental details were thoroughly explained to all participants, who then provided their consent by signing a consent form. All participants completed the study. However, a male participant did not record the preset running time; thus, this participant's data was treated as missing in the analysis of preset running time and speed. The study was conducted according to the guidelines of the Declaration of Helsinki and was approved by the Ethics Committee of the Juntendo University Faculty of Health and Sports Science (approval number: 23‐37).

**TABLE 1:** Characteristic of participants.

	Male	Female
	(*n* = 25)	(*n* = 17)
	Mean ± SD	Mean ± SD
Age (years)	20.0 ± 0.6	19.9 ± 0.4
Height (cm)	174.8 ± 5.8	158.5 ± 3.3
Body weight (kg)	68.8 ± 10.8	50.2 ± 2.3
BMI (kg/m^2^)	22.5 ± 3.2	20.0 ± 1.1
AMY1 Copy Number^a^	6 (2–16)	5 (2–12)

^a^ Median (Range).

### 2.2 Study design

Participants assembled in the gymnasium at 12:50 in the afternoon. without having consumed lunch. They lay down and listened to relaxing music for 10 min to ensure a restful state. Saliva samples were then collected pre‐exercise using Salisoft® (Zalstat, Tokyo, Japan). Each participant performed a self‐chosen warm‐up pre‐exercise. Participants were paired up, with each pair performing the exercise test twice, while one participant recorded the other's performance.

**FIGURE 1:**
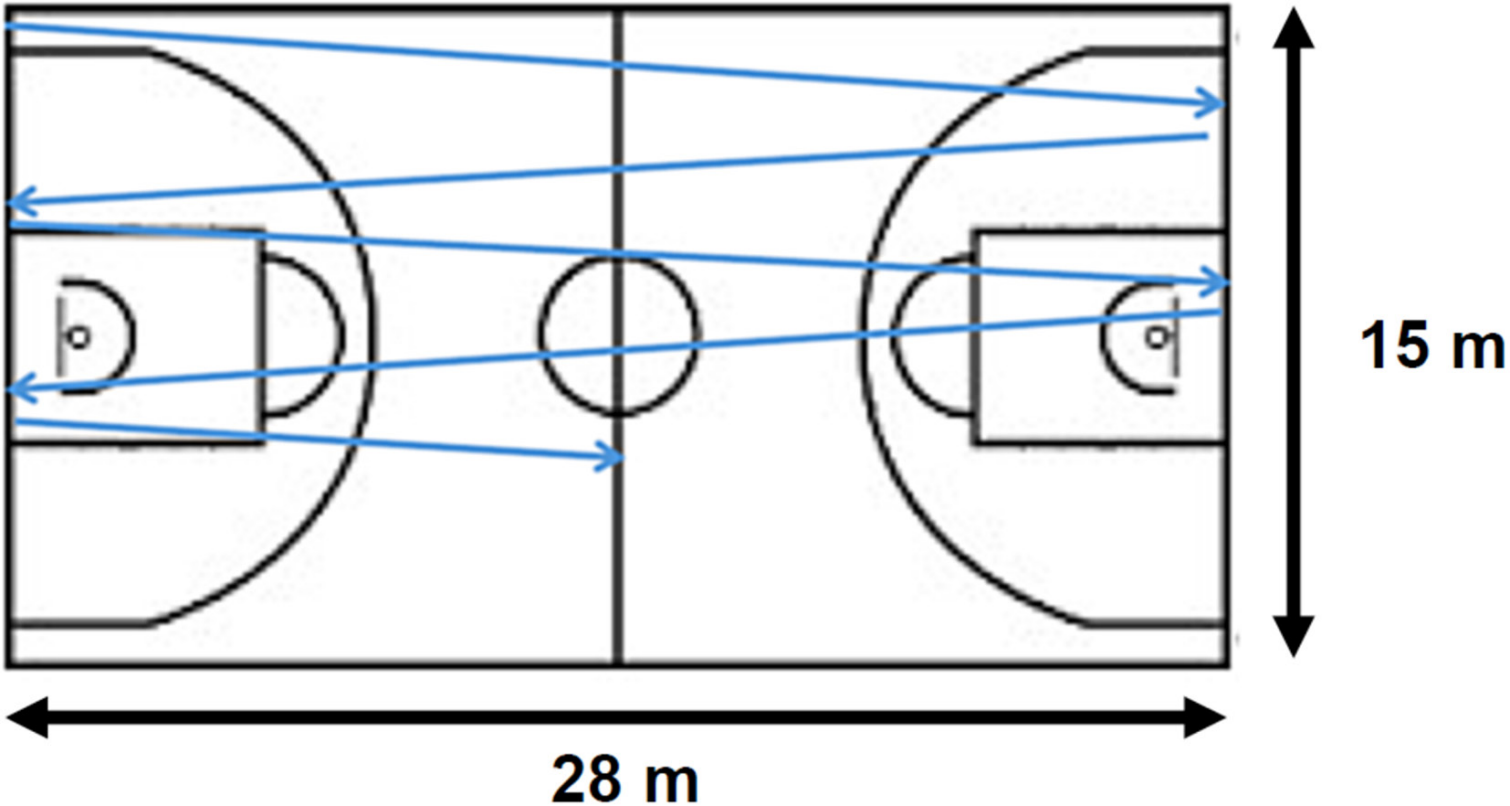
Schematic representation of the interval training protocol. The blue arrow illustrates two and a quarter round‐trips around the basketball court. In practice, participants run vertically from one end of the court to the other.

The selected exercise protocol was an interval training method developed by the Kanto Collegiate Basketball Federation (Shimizu et al., 2009). This protocol, described in a Japanese basketball journal in April 2009, is widely used in the Japanese basketball community. Briefly, participants ran intermittently for two and a quarter repetitions along the long side of the basketball court, the total distance of 126 m (Figure 1). Starting from the end line on one side of the court, participants ran to the opposite end line, touched it, then turned back and ran to the original end line. This was done for two consecutive laps and up to the halfway line. The running time was preset to range from 24 to 30 s (speed of 4.2–5.25 m/sec), depending on their aerobic fitness. After reaching the halfway point, participants moved to the finish line on the other side of the court before the next start. The interval between starts was 90 s. Participants performed this exercise 10 times or until they could no longer complete the runs within the preset time.

Saliva was collected before and immediately after the exercise test. The collected saliva was immediately cooled on ice, then centrifuged, frozen, and stored until analysis.

Participants reconvened in the laboratory at 14:40 to collect oral mucosa samples. Mucosal cells were collected using a cotton swab, which was placed on the inside of the cheek and rubbed approximately 10 times in a rotating motion. The swab, containing the mucosal cells, was then placed in a 1.5 mL microtube with 300 μL of extraction buffer from the ISOHAIR DNA extraction kit (cat. No. 319‐3401, Nippon Gene, Tokyo, Japan). The swab was swirled several times to remove as much fluid as possible, and the mucosal cells were harvested.

### 2.3 Salivary amylase activity

Amylase activity and total protein concentration of the collected saliva were measured by the clinical laboratory SRL (Tokyo, Japan). Amylase activity was assessed using a standard automated method recommended by the Japanese Committee for Clinical Laboratory Standards, while total protein was measured using the Biuret method. Amylase activity was expressed as U/mg protein.

### 2.4 AMY1 copy number

DNA extraction from the ISOHAIR Extraction Buffer was performed according to the manufacturer's protocol. Briefly, 10 μL of enzyme solution (Proteinase K) and 8 μL of lysis solution were added to 180 μL of extraction solution and mixed at 55°C for 20 min. The mixture was then incubated at 55°C for 20 min. Subsequently, 5 μL of lysis solution was added, mixed, and incubated at 55°C for 10 min. After incubation, 200 μL of phenol/chloroform was added, and the mixture was covered, inverted, and mixed for 5 min. After centrifugation at 12,000 rpm for 5 min at 4°C, 200 μL of the aqueous phase was collected with a pipette. To this, 20 μL of 3 M sodium acetate and 2 μL of ethachinmate were added and mixed. Then, 400 μL of absolute ethanol was added, mixed, and centrifuged at 12,000 rpm for 15 min at 4°C to precipitate the DNA. The resulting DNA was washed with 500 μL of 70% ethanol and then dissolved in 20 μL of TE buffer.

AMY1 copy number was estimated using real‐time polymerase chain reaction (PCR) on an Applied Biosystems 7500 System as reported previously (Iafrate et al., 2004). The PCR reagents were SYBR Green ER qPCR Super Mix Universal (cat. no. 1176202K, Invitrogen, Thermo Fisher Scientific, Tokyo, Japan). The FOXP2 gene served as a control for AMY1. The extracted chimpanzee genome DNA solution, provided by the Primate Research Institute, Kyoto University, which contains a single copy of the AMY1 gene per genome set (2 copies/diploid), was used as the standard for calibration. The chimpanzee standard genome was prepared as a 6‐step dilution series. Real‐time PCR reaction conditions were 50°C for 2 min, 95°C for 10 min, followed by 40 cycles of 95°C for 15 s and 60°C for 60 s.

AMY1 copy number was calculated by comparing with FOXP2 using the relative quantification method. Briefly, with the cycle threshold (Ct) as the explanatory variable, calibration curves for AMY1 and FOXP2 were constructed using a logarithmic dilution series (6 steps at twofold intervals) of the chimpanzee genome standard solution. The calibration curve was accepted if the coefficient of determination was ≥0.9. When the sample Ct fell within the calibration curve range, the relative amounts of AMY1 and FOXP2 were measured. The AMY1 copy number was estimated from the ratio of AMY1 to FOXP2, rounded to one decimal place. All measurements were performed in triplicate, and only those with a coefficient of variation of less than 5% for the triplicate data and relative AMY1 values within the calibration curve were accepted. Samples not meeting these criteria were retested.

### 2.5 Statistical analysis

The normality of each continuous variable was assessed using the Shapiro–Wilk test. As normality could not be assumed for running distance in the exercise test, the Mann–Whitney test was used to compare means between males and females. For amylase activity, which was normally distributed, mean differences between male and female were compared by Student's *t*‐test, and pre‐ and post‐exercise means were compared using the paired *t*‐test. Since AMY1 copy number did not follow a normal distribution, the Spearman correlation coefficient was used to evaluate its correlation with amylase activity. The power of Spearman's correlation coefficient was calculated based on effect size (*r*), sample size (*n*), and a significance level of 0.05.

The relationship between the change in salivary amylase activity per AMY1 gene copy and AMY1 copy number was analyzed using a generalized linear model, adjusting for possible confounders: sex, age, BMI, and preset running speed during interval training. All 16 combinations of these variables were tested—with and without each variable as a predictor—in a model in which the change in salivary amylase activity per copy of the AMY1 gene and AMY1 copy number were the response and predictor variables, respectively. Model fit was evaluated using Akaike's Information Criterion (AIC), with lower values indicating better fit. Additionally, male and female pre‐ and post‐exercise amylase activities were compared using generalized linear model controlled for AMY 1 copy number and preset running time.

The data were presented as mean ± SD, and generalized linear model results were marginal means ± standard error. Statistical analysis was performed using the Statistical Package for Social Sciences version 29 (IBM Japan, Tokyo, Japan), with a significance level set at less than 5%.

## 3 RESULTS

### 3.1 Running distance in exercise test

The mean distance run during the exercise test was 1003 ± 312 meters for males and 645 ± 396 meters for females, with males covering a significantly longer distance (*p* = 0.005).

### 3.2 Amylase activity

Salivary amylase activity before the exercise test was 95.6 ± 49.5 U/mg protein. It was significantly higher in males (109.0 ± 53.6 U/mg protein) compared to females (75.7 ± 34.6 U/mg protein) (*p* = 0.03).

Transient exercise significantly increased salivary amylase activity to 141.5 ± 43.8 U/mg protein (*p* < 0.001). Amylase activity increased in 37 of the 42 participants. Among the five participants whose amylase activity did not increase after exercise, the mean distance run was 1108.8 ± 225.4 meters, which was not significantly different (*p* = 0.12) from the 824.1 ± 393.6 meters run by the other participants; however, three of these five participants reached the upper limit of 1260 m.

There was no significant difference in amylase activity after exercise between males (147.3 ± 48.1 U/mg protein) and females (133.1 ± 35.4 U/mg protein). Additionally, the changes in amylase activity from pre‐ to post‐exercise did not differ significantly between males (38.2 ± 44.6 U/mg protein) and females (57.4 ± 43.5 U/mg protein) (Table 2).

**TABLE 2:** Summary of amylase activity.

	Pre‐exercise	Postexercise	*p* Value	Change
	Mean ± SD	Mean ± SD		Mean ± SD
Total	95.6 ± 49.5	141.5 ± 43.8	<0.001	46.0 ± 44.6
Male	109.0 ± 53.6	147.3 ± 48.1	<0.001	38.2 ± 43.7
Female	75.7 ± 34.6	133.1 ± 35.4	<0.001	57.4 ± 43.5
*p* value (male vs. female)	0.030	0.309		0.176

*Note*: Amylase activity (U/mg protein).

### 3.3 AMY1 copy number

**FIGURE 2:**
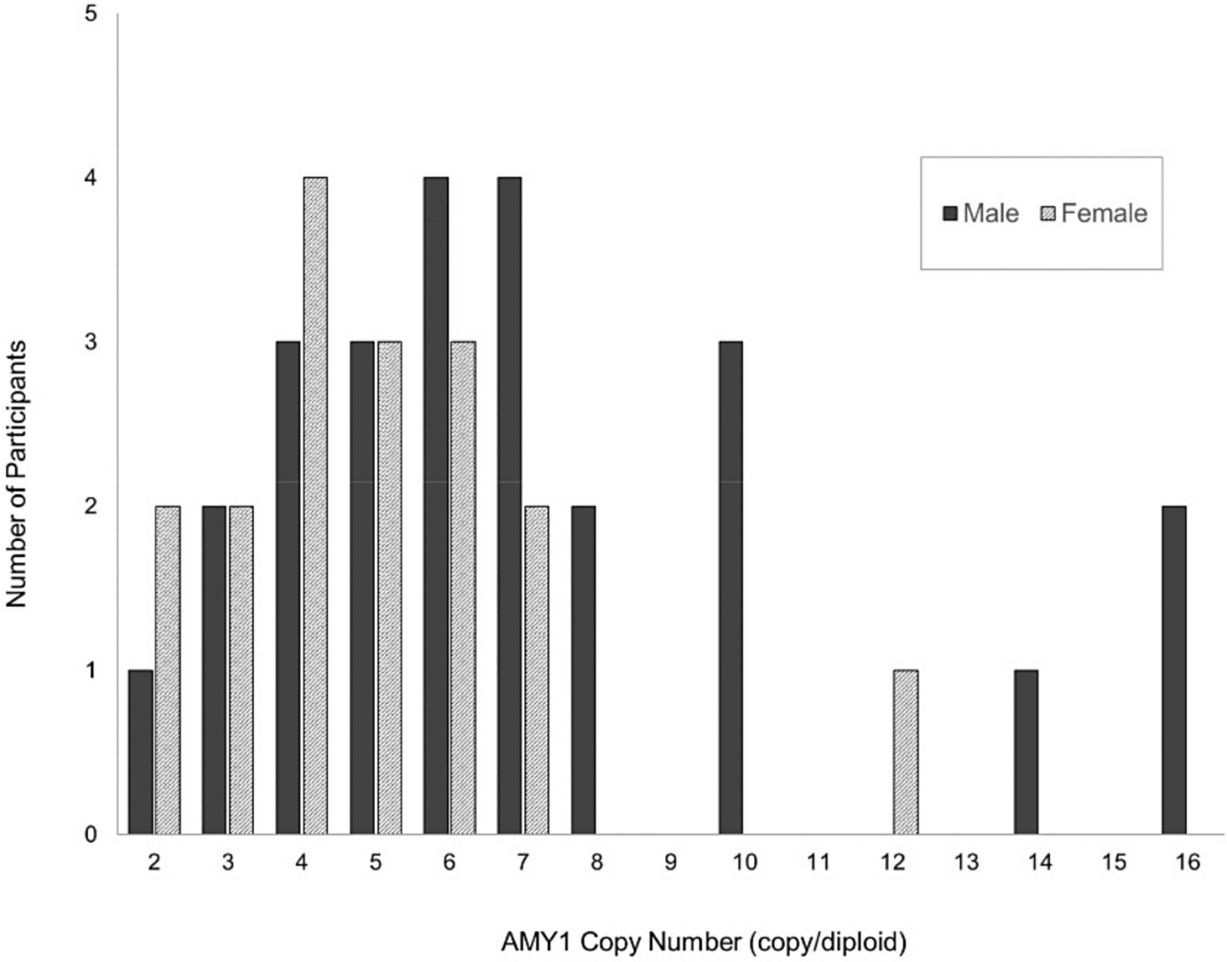
Distribution of AMY1 copy number. Horizontal axis represents the number of AMY1 gene copies, and the vertical axis shows the number of participants. The median AMY1 copy number was 6 for men and 5 for women.

The median (range) of AMY1 copy number was significantly higher in males (6, (2, 16)) compared to females (5 (2, 12), *p* = 0.03) (Figure 2).

### 3.4 AMY1 copy number and salivary amylase activity

**FIGURE 3:**
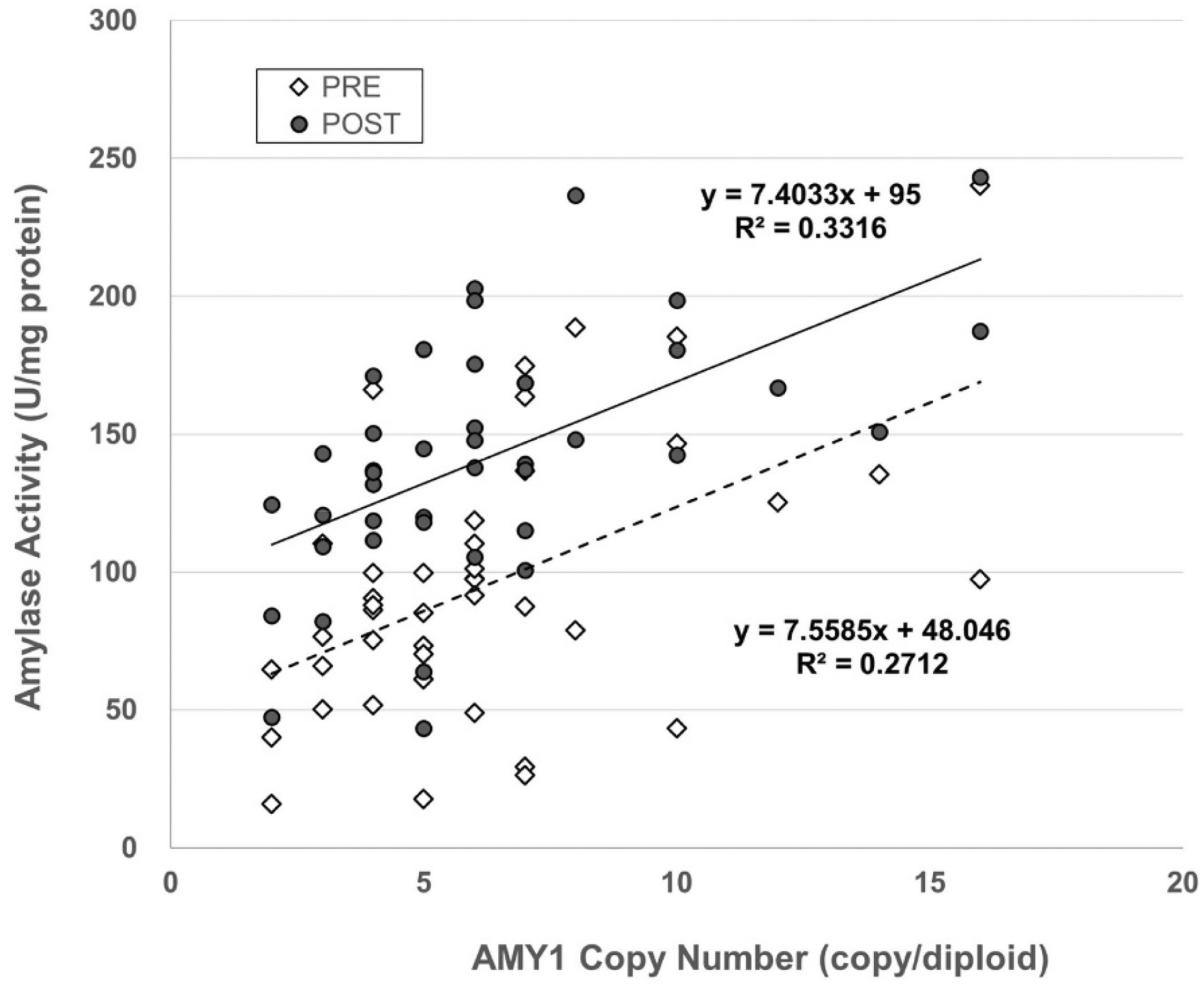
Relationship between AMY1 copy number and salivary amylase activity pre‐ and post‐exercise. The horizontal axis shows the number of copies of the AMY1 gene, and the vertical axis shows the amylase activity. Open and closed symbols showed pre‐ and post‐exercise, respectively.

AMY1 copy number showed significant positive correlations with salivary amylase activity before (*r* = 0.476, *p* < 0.01, power = 0.865) and after (*r* = 0.586, *p* < 0.01, power = 0.972) the exercise test (Figure 3). When AMY1 copy number was denoted as x and amylase activity as y, the regression lines for pre‐ and post‐exercise were *y* = 7.56*x* + 48.05 (*R* ^2^ = 0.271) and *y* = 7.40*x* + 95.00 (*R* ^2^ = 0.3316), respectively.

### 3.5 AMY1 copy number and exercise‐induced change in amylase activity

**FIGURE 4:**
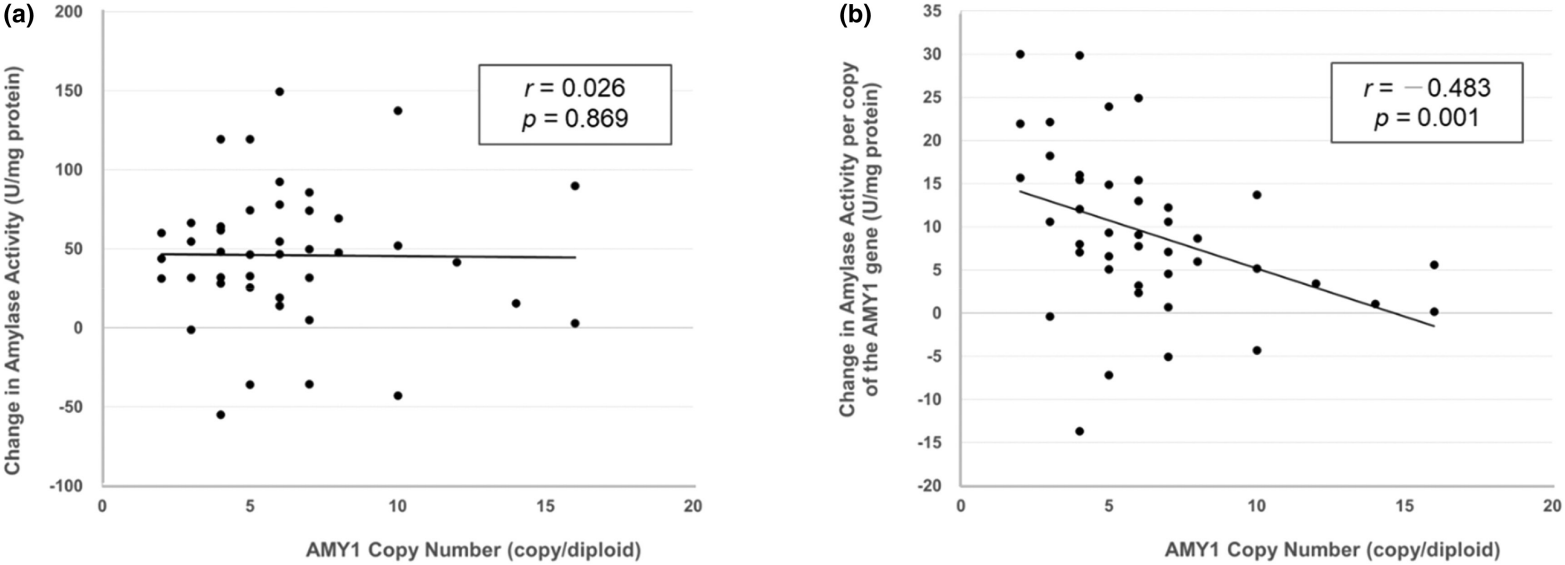
Relationship of AMY1 copy number and the change in exercise‐induced change in salivary amylase activity. Horizontal axis represents the number of AMY1 gene copies, and the vertical axis shows (a) the change in salivary amylase activity and (b) the change per AMY1 gene copy.

The change in amylase activity induced by exercise had almost no relationship with the AMY1 copy number (*r* = 0.026, *p* = 0.336, power = 0.053), whereas the change per copy of the AMY1 gene showed a significant negative correlation with AMY1 copy number (*r* = −0.483, *p* < 0.001, power = 0.876) (Figure 4).

The exercise‐induced change in salivary amylase activity per AMY1 gene was significantly greater in females (13.0 ± 10.3 U/mg protein) than in males (6.9 ± 8.2 U/mg protein) (*p* = 0.040). This change showed a significant negative correlation with the preset running speed during interval training (*r* = −0.458, *p* = 0.003, power = 0.826); however, no significant correlations were found with running distance (*r* = −0.216, *p* = 0.169, power = −0.273), age (*r* = 0.192, *p* = 0.223, power = 0.225), or BMI (*r* = 0.024, *p* = 0.880, power = 0.053). To account for potential confounders in the relationship between the change in salivary amylase activity per AMY1 gene copy and the AMY1 copy number, all combinations of the four variables—preset running speed, sex, age, and BMI—were analyzed using a generalized linear model.

The best‐fitting model (with the lowest AIC) included AMY1 copy number and preset running speed as predictor variables (Model 1), followed by the model with AMY1 copy number and sex as predictor variables (Model 2). These two models fit better than the model with AMY1 copy number as the only predictor variable (Model 3). In all models (1, 2, and 3), the regression coefficient for AMY1 copy number was significantly negative. In addition, preset running speed (Model 1) was significantly negative, and sex (Model 2) was not significant (Table 3). The AIC and the combinations of predictor variables for the 16 models examined are summarized in Table S1.

**TABLE 3:** Top three models predicting the exercise‐induced change in salivary amylase activity per AMY1 gene.

Model	AIC	*p* ^a^	Parameter	B	Std. error	*p* ^b^
1	297.821	0.004	Intercept	50.848	17.445	0.004
			AMY1 copy number	−0.822	0.407	0.044
			preset running speed	−8.010	4.005	0.045
2	299.616	0.010	Intercept	17.586	2.903	<0.001
			AMY1 copy number	−0.927	0.410	0.024
			Sex = Male	−4.082	2.835	0.150
			Sex = Female	0^c^		
3	299.638	0.007	Intercept	16.381	2.849	<0.001
			AMY1 copy number	−1.115	0.398	0.005

^a^ *p* for the model.

^b^ *p* for the parameter.

^c^ Set to zero because this parameter is redundant.

### 3.6 Aerobic fitness and gender on amylase activity

We examined the relationship between preset running speed and salivary amylase activity. Reportedly, the activity was significantly correlated with preset running speed pre‐exercise (*r* = 0.364, *p* = 0.019, power = 0.624); however, the correlation was not significant postexercise (*r* = 0.130, *p* = 0.418, power = 0.126).

As the change in amylase activity per copy of the AMY1 gene showed a significant sex difference, as well as significant correlations with AMY1 copy number and preset running speed, it was compared by sex using a generalized linear model controlled for AMY1 copy number and preset running speed. The estimated marginal means of the change did not differ between males (9.7 ± 2.4 U/mg protein) and females (8.9 ± 3.1 U/mg protein) (*p* = 0.855). Amylase activity did not show any significant sex difference when controlled for AMY1 copy number and preset running speed, either pre‐ (*p* = 0.257) or post‐exercise (*p* = 0.093) (Table S2).

## 4 DISCUSSION

Herein, salivary amylase activity was positively correlated with AMY1 copy number pre‐ and post‐exercise and increased in response to exercise. Aerobic fitness was also positively correlated with the activity. The exercise‐induced change in the activity was almost independent of the AMY1 copy number. In contrast, the change per AMY1 gene was negatively related to the AMY1 copy number and aerobic fitness. Gender has no effect on amylase activity.

The human AMY1 gene, located on chromosome 1 (1p21.1), is known for its high copy number variation (Perry et al., 2007). Notably, the salivary amylase gene includes three similar genes: AMY1A, AMY1B, and AMY1C (Samuelson et al., 1996). However, the number of AMY1 copies in humans ranges from 2 to 20, with 90% of individuals having between 4 and 12 copies (Al‐Akl et al., 2020; Alberti et al., 2015; Falchi et al., 2014; Perry et al., 2007). Since AMY1 is located on an autosome rather than a sex chromosome, there is no inherent sex difference in copy number (Al‐Akl et al., 2020; Falchi et al., 2014). In this study, the median AMY1 copy number was similar for males and females (6 vs. 5). However, the mean copy number was statistically higher in males due to the presence of one male with 14 copies and two others with 16 copies. This discrepancy is not indicative of a gender difference but rather reflects the small sample size of 25 males and 17 females.

AMY1 copy number is significantly correlated with salivary amylase activity; higher AMY1 copy numbers are associated with increased amylase activity (Alberti et al., 2015; Carpenter et al., 2017; Falchi et al., 2014; Mandel & Breslin, 2012; Perry et al., 2007). In this study, AMY1 copy number and salivary amylase activity were positively correlated before and after the exercise test. Pre‐exercise amylase activity was higher in males compared to females, reflecting the higher AMY1 copy number in males. However, no sex difference was observed in the exercise‐induced change in amylase activity. Consequently, data from males and females were combined for evaluation. This study also confirmed the association between AMY1 copy number and salivary amylase activity.

Amylase activity significantly increases in response to high‐intensity exercise such as at or above 70% VO_2_max (Koibuchi & Suzuki, 2014). Recent studies have also reported increases in salivary amylase activity following a single bout of high‐intensity Rating of Perceived Exertion (RPE)‐based cycling exercise (Weiss et al., 2019). In this study, salivary amylase activity increased as participants performed 2.25 round‐trip runs to the limit of their endurance, despite variations in running distance. This increase was observed in 37 of 42 participants. Therefore, this study confirmed that transient intense exercise effectively enhances salivary amylase activity and that the exercise test provided a sufficiently intense load to impact amylase activity.

To date, many studies have reported the exercise‐induced increase in amylase activity, while the relationship between the increases and AMY1 copy number has not been studied at all. In this study, the changes in activity (postexercise minus pre‐exercise) were almost independent of AMY1 copy number but had a significant negative correlation with participants' aerobic fitness. Therefore, studies on exercise‐induced changes in salivary amylase activity are considered valid, even if AMY1 copy number are not examined, provided the participants have equivalent aerobic fitness.

Reportedly, sustained physical training reduces changes in blood catecholamines in response to exercise (Hackney, 2006). Additionally, 12 weeks of exercise significantly flattens the diurnal cortisol slope and reduces cortisol levels upon awakening (Rahman et al., 2019). However, such training adaptations have not been reported for salivary amylase. Herein, the change in amylase activity per AMY1 gene was negatively correlated with AMY1 copy number and aerobic fitness. Specifically, the greater the number of AMY1 copies, the smaller the change in activity, and the greater the preset running speed (reflecting aerobic capacity), the smaller the change in activity.

The relationship between aerobic fitness and pre‐exercise salivary amylase activity along with exercise‐induced changes in amylase activity may reflect training adaptations. The finding that AMY1 copy number is positively correlated with amylase activity pre‐exercise but negatively correlated with exercise‐induced increases in activity per AMY1 gene suggests a potential difference between constitutive and exercise‐induced amylase secretion. Further research is needed to explore the underlying mechanisms.

However, the abovementioned findings may be influenced by the small sample size, as only seven participants (17% of the total) had an AMY1 copy number of 10 or greater. Therefore, further studies including more high copy number participants are needed to better understand the effect of AMY1 copy number on exercise‐induced changes in salivary amylase activity.

Males generally have a higher blood volume and aerobic exercise capacity compared to females (Diaz‐Canestro et al., 2022; Lundby & Robach, 2015). Therefore, applying the same aerobic load across a mixed‐gender population using a single protocol can be challenging. The interval training used in this study involved running up to a maximum of 10 runs or until the exercise could not be completed within the preset time. Males ran a significantly longer total distance than females. However, there were no significant differences between males and females in pre‐ and post‐exercise amylase activity or in the change in response to exercise when controlled for AMY1 copy number and aerobic fitness. Therefore, the interval training protocol used in this study may be effective for providing a consistent level of aerobic exercise across different genders or aerobic capacities.

There were several limitations in this study. First, individual aerobic capacity was not considered before the exercise test, allowing participants to set their preset running times. Additionally, participants could terminate the exercise test at their discretion, which may have led some to end the test prematurely. The maximum number of interval runs was set at 10, which may have been insufficient for participants with high aerobic capacity, limiting the maximum total distance to 1260 m. Because aerobic exercise capacity is related to salivary amylase activity, future studies should confirm this relationship by directly measuring the aerobic exercise capacity of the participants. Another limitation was the small sample size, with 25 males and 17 females, leading to an uneven number of participants across genders. Further studies with a larger sample size are needed to validate our findings. To better understand the relationship of salivary amylase activity with AMY1 copy number and aerobic fitness, future studies would benefit from including gene expression analyses. Investigating AMY1 gene expression levels, in addition to copy number, could offer comprehensive insights into how genetic variations influence amylase activity and its response to exercise.

## 5 CONCLUSION

Human salivary amylase activity was positively correlated with AMY1 copy number and increased in response to exercise. The exercise‐induced change in amylase activity was independent of AMY1 copy number or sex but negatively correlated with the aerobic fitness of participants. Additionally, AMY1 copy number was negatively correlated with exercise‐induced increases in activity per AMY1 gene. Despite the widespread use of amylase activity as an indicator for various stresses, no previous studies have investigated its relationship with AMY1 copy number. The findings of this study suggest that conclusions from studies on exercise‐induced changes in amylase activity using participants with equivalent aerobic fitness can be considered valid, even if AMY1 copy number was not examined. However, studies using salivary amylase activity as a stress indicator in other contexts should be re‐evaluated, considering AMY1 copy number.

## AUTHOR CONTRIBUTIONS

YK was involved in writing—review and editing, writing—original draft, investigation, methodology, and visualization. EK was involved in writing—review and editing, writing—original draft, investigation, methodology, and data curation. KS was involved in writing—review and editing and project administration. YS was involved in writing—review and editing, conceptualization, formal analysis, funding acquisition, methodology, project administration, resources, supervision, and visualization.

## FUNDING INFORMATION

This work was supported by the Research Encouragement Program of Juntendo University, Faculty of Health and Sports Science.

## CONFLICT OF INTEREST STATEMENT

The authors declare no conflicts of interest.

## ETHICS STATEMENT

The study was conducted according to the guidelines of the Declaration of Helsinki and was approved by the Ethics Committee of the Juntendo University Faculty of Health and Sports Science (approval number: 23‐37).

## Supporting information


Tables S1–S2.

## Data Availability

Data are available upon request to the corresponding author.
