# Supplementary material for: Influence of AMY1 gene copy number on salivary amylase activity changes induced by exercise in young adults
Source: Physiol Rep. 2024 Oct 25;12(20):e70099. doi: 10.14814/phy2.70099 (PMC11503727; doi:10.14814/phy2.70099)
Supplement: Supplementary file 1 — Tables S1–S2. [file PHY2-12-e70099-s001.pdf]

Supplementary Table 1: Predictive variables and Akaike's Information Criterion (AIC) for models examined using generalized linear models

Model	Predicators					AIC
	AMY1 copy number	Sex	Age	BMI	Preset running speed	
1	+				+	297.821
2	+	+				299.616
3	+					299.638
4	+		+		+	299.714
5	+			+	+	299.726
6	+	+			+	299.794
7	+	+		+		301.377
8	+	+	+			301.440
9	+			+		301.550
10	+		+			301.583
11	+		+	+	+	301.675
12	+	+	+		+	301.701
13	+	+		+	+	301.725
14	+	+	+	+		303.310
15	+		+	+		303.410
16	+	+	+	+	+	303.674

+, included in the model.

Supplementary Table 2: Male and female salivary amylase activity controlled for AMY1 copy number and preset running time

Dependent variable	Parameter	B	Std. Error	*p*		EMM	Std. Error	*p*
Amylase activity (pre-exercise)	Intercept	91.3	134.1	0.496				
	Sex = Male	25.3	22.3	0.257		105.9	11.3	0.257
	Sex = Female	0[a]				80.6	14.6	
	AMY1 copy number	6.8	2.0	<0.001	***			
	Preset running speed	−11.8	32.2	0.713				
Amylase activity (postexercise)	Intercept	346.6	109.5	0.002	**			
	Sex = Male	30.5	18.2	0.093		154.0	9.2	0.093
	Sex = Female	0[a]				123.5	11.9	
	AMY1 copy number	8.2	1.7	<0.001	***			
	Preset running speed	−60.6	26.3	0.021	*			

EMM, estimated marginal mean; [a], set to zero because this parameter is redundant.

* $p < 0.05$, ** $p < 0.01$, *** $p < 0.001$
